# Correlation and risk factor analysis of suicidal behavior in adolescents with depression: the impact of stress and childhood trauma

**DOI:** 10.3389/fpsyt.2025.1567071

**Published:** 2025-07-30

**Authors:** Lilei Dai, Hui Du, Dan Zhao, Jing Shu, Xinfu He

**Affiliations:** Department of Clinical Psychology, JingMen People’s Hospital, Jingchu University of Technology Affiliated JingMen People’s Hospital, Jingmen, China

**Keywords:** adolescent depression, suicide, stressors, childhood abuse, risk factors

## Abstract

**Objective:**

This study investigates the correlation between suicidal behavior and various stress factors in a sample of hospitalized adolescents with depression.

**Methods:**

A cross-sectional survey was conducted on 254 adolescent patients with depression from Aug 2022 to May 2024. Participants were assessed using PHQ-11, CTQ-SF, and SSMSS.

**Results:**

The suicide group (150 cases) had higher stress levels, more severe childhood trauma, and a higher prevalence of females, alcohol consumption, and lower age compared to the non-suicide group (104 cases). Risk factors included lower age, poor interpersonal relationships, emotional abuse, and alcohol consumption.

**Conclusion:**

The study highlights the need to address stress factors and provide psychological support to safeguard adolescents’ mental health.

## 1 Introduction

The increasing prevalence of depression in the context of economic and societal development has emerged as a critical public health concern (1), particularly among adolescents. The “China National Mental Health Development Report” in 2020 highlighted a startling statistic: the detection rate of depressive mood among Chinese adolescents is 24.6%, with severe depressive mood affecting 7.4% of this population (2). Adolescents with depression constitute 30.28% of all patients with depression, and a significant proportion, 50%, are students. The incidence rate of depression in Chinese adolescents is alarmingly high, ranging from 15% to 20%. Self-harm and suicidal ideation are common symptoms within this demographic, with suicide emerging as a leading cause of death among adolescents, a trend that has been escalating in recent years (3). Adolescent depression is a complex interplay of biological and environmental factors, with the latter playing a pivotal role in the genesis of depression and suicidal behaviors (4). The severity of this issue is further underscored by a meta-analysis encompassing 686,672 children and adolescents worldwide, which suggests that the lifetime and one-year suicide rates for this age group are 18.0% and 14.2%, respectively (5), from 1989 to 2018. Our previous research (6) indicated that 60% of adolescents with depression exhibit self-harm and suicidal behaviors, Despite the recognized importance of environmental factors in the development of depression and suicidal behaviors (7–9), the specific components of these environmental factors and their relationships with the progression of depression or the emergence of suicidal behaviors have not been extensively studied. Understanding these relationships is crucial for developing targeted interventions and policies aimed at protecting the mental health of adolescents.

This study aims to investigate the correlation between suicidal behavior and various stress factors in adolescents with depression, using a sample of hospitalized patients. Future research should consider broader samples to enhance generalizability. By examining the nexus between depression and a spectrum of stressors, including childhood trauma and adolescent stress, we aim to expand the knowledge base on adolescent mental health and inform the development of targeted preventive measures. The significance of this research is manifold, as it not only seeks to identify at-risk populations and enhance early intervention efforts but also aspires to reduce the incidence of suicidal behavior among adolescents with depression. Through a comprehensive analysis of risk factors, this study aims to provide valuable insights into the multifaceted nature of adolescent depression and the pivotal role of environmental stressors in the development of suicidal tendencies.

## 2 Methods

### 2.1 Study subjects

This study employed a cross-sectional survey method, selecting adolescents hospitalized in the Clinical Psychology Department of Jingmen People’s Hospital from Aug 2022 to May 2024 as the study subjects. The sample was limited to hospitalized patients, which may introduce selection bias. Future studies should consider including outpatients or community-based adolescents to enhance generalizability. Inclusion criteria: (1) All met the diagnostic criteria for depressive episodes of affective disorders according to the 11th edition of the International Classification of Diseases (ICD-11); (2) Diagnosed by a resident physician and an associate chief physician or above; (3) Age 11-18, both genders included. Exclusion criteria: (1) History of severe organic diseases; (2) Depression caused by other psychoactive substances; (3)Suffering from other mental disorders besides depression; (4)Patients with severe cognitive dysfunction; (5) Patients with vision or hearing impairments; (6) Patients with poor communication and understanding, making psychological assessment difficult; (7) Patients with incomplete data due to missed questions on the scale. Suicidal behavior was defined as patients having subjective intent to commit suicide, engaging in deliberate self-destructive behavior, and a score of ≥12 on the Suicide Ideation Self-Rating Scale (SIOSS). However, this definition conflates suicidal ideation with actual behavior. Future studies should use more validated criteria, such as the Columbia-Suicide Severity Rating Scale (C-SSRS), to distinguish between suicidal ideation and behavior.

The study surveyed 260 subjects, with 254 valid samples, a validity rate of 97.7%. According to whether there was suicidal behavior, the subjects were divided into a suicide group of 150 cases and a non-suicide group of 104 cases. Patients and their families voluntarily participated and signed informed consent forms. This study was approved by the hospital’s ethics committee (approval number 2022071903).

### 2.2 Research tools

1. General survey form: Including age, gender, ethnicity, grade, place of residence, only child, smoking and drinking history, economic status, family history of mental illness, family situation, and whether there has been suicidal behavior in the past 6 months, etc.
2. Patient health questionnaire-9 (PHQ-9) (10): This scale consists of 10 items, including 9 symptom scales and 1 functional total score, assessing the depressive mood of the tester within 2 weeks. The scale uses a 0-3 point 4-grade scoring method, with a total score of less than 4 points indicating no depressive mood, 5-9 points indicating mild depression, 10-14 points indicating moderate depression, and ≥15 points indicating severe depression.
3. Stressors scale for middle school students (SSMSS) (11): Developed by Zheng Quanquan, Chen Shulin, and Zheng Shengsheng in 1999, it includes 7 factors: learning pressure, teacher pressure, family environment pressure, parental discipline pressure, classmate and friend pressure, social and cultural pressure, and self-physical and mental pressure, with a total of 39 items. Respondents are asked to recall whether the events described in the scale have occurred in the past year and at the moment, and how much impact these events have had on them.
4. Childhood trauma questionnaire-short form (CTQ-SF) (12): This questionnaire includes 5 dimensions: emotional abuse, physical abuse, sexual abuse, emotional neglect, and physical neglect, with a total of 28 items, using a 5-point scoring method. The higher the score, the more severe the trauma. If the emotional abuse subscale total score is ≥13 points, it indicates a history of childhood emotional abuse; if the physical abuse subscale total score is ≥10 points, it indicates a history of physical abuse; if the sexual abuse subscale total score is ≥8 points, it indicates a history of sexual abuse; if the emotional neglect subscale total score is ≥15 points, it indicates a history of emotional neglect; if the physical neglect subscale total score is ≥10 points, it indicates a history of physical neglect. If at least one of the 5 factors is present, the study subject is determined to have a history of childhood trauma.

### 2.3 Statistical analysis

Data was statistically analyzed using the SPSS22.0 software package. Quantitative data conforming to a normal distribution was expressed as ( ± s) and analyzed using independent sample t-tests. Quantitative data not conforming to a normal distribution was expressed as M(P25, P75) and analyzed using non-parametric tests; count data was expressed as the number of cases and analyzed using Chi-square tests. Suicide risk factor analysis was performed using Spearman correlation and logistic regression analysis. To address multiple comparisons, a Bonferroni correction was applied, adjusting the significance level accordingly. Additionally, variance inflation factors (VIF) were calculated to assess multicollinearity in the regression models.

## 3 Results

### 3.1 Comparison of general data between the two groups

There were no significant differences in grade, place of residence, only child status, smoking, family history of mental illness, economic status, and family situation between the two groups (P>0.05). The age of the suicide group was significantly lower than that of the non-suicide group (P=0.005). The proportion of females was higher in the suicide group (P=0.032), and the rate of alcohol consumption was higher (P=0.020). However, the binary assessment of alcohol consumption does not account for frequency or severity, which may limit the interpretation of this finding, as shown in **Table 1**.

**Table 1:** Comparison of general data between the two groups ( ± s, number of cases).

Item		Suicide group (N=150)	Non-suicide group (N=104)	t/X^2^	P
Age		14.48 ± 1.71	15.10 ± 1.67	-2.285	0.005
Gender	Male	25	29	4.617	0.032
	Female	125	75		
Grade	Junior Grade 1	29	13	11.778	0.067
	Junior Grade 2	25	14		
	Junior Grade 3	34	14		
	Senior Grade 1	20	24		
	Senior Grade 2	23	16		
	Senior Grade 3	12	16		
	other	7	7		
Residence	Urban	96	54	0.005	0.945
	Rural	67	37		
Only Child	YES	88	67	0.856	0.355
	NO	62	37		
Smoking	YES	19	8	1.600	0.206
	NO	131	96		
Drinking	YES	52	22	5.432	0.020
	NO	98	82		
Family History	YES	36	114	2.179	0.140
	NO	17	87		
Economic Status	Very Poor	4	3	2.752	0.600
	Fair	25	16		
	Average	105	79		
	Good	14	6		
	Excellent	2	0		
Family Situation	Nuclear Family	109	84	2.270	0.321
	Reconstituted Family	13	7		
	Single-Parent Family	28	13		

### 3.2 Comparison of SSMSS scores between the two groups

The total score of SSMSS in the suicide group was higher than in the non-suicide group, reflecting higher scores in factors such as teacher pressure, family environment, parental upbringing, friends and classmates, social culture, and self-physical and mental aspects, with significant differences between the two groups (P<0.05), as shown in **Table 2**.

**Table 2:** Comparison of total and factor scores of SSMSS between the two groups [M(P25,P75)].

Item	Suicide group (N=150)	Non-suicide group (N=104)	Z	P
SSMSS Total Score	61.0 (47.0,80.5)	51.0 (37.3,73.3)	-3.360	<0.01
Learning Pressure	11.0 (8.0,15.0)	12.0 (7.3,14.0)	-0.034	0.973
Teacher Pressure	9.5 (5.0,15.0)	8.0 (2.3,13.0)	-2.431	0.015
Family Environment	6.0 (3.0,10.3)	4.5 (2.0,8.0)	-2.874	<0.01
Parental Upbringing	5.5 (3.0,9.0)	4.0 (6.0,15.0)	-2.706	<0.01
Friends and Classmates	13.0 (8.0,17.0)	9.0 (6.0,15.0)	-3.227	<0.01
Social Culture	5.0 (2.8,9.0)	4.0 (1.0,6.0)	-2.924	<0.01
Self-Physical and Mental	11.0 (8.0,14.0)	9.5 (7.0,12.0)	-3.329	<0.01

### 3.3 Comparison of CTQ scores between the two groups

The total score of CTQ and all factor scores in the suicide group were higher than in the non-suicide group, with significant differences between the two groups (P<0.05), as shown in **Table 3**. The total score of SSMSS and CTQ in the suicide group was higher than in the non-suicide group (P<0.05). However, the lack of a healthy control group limits the ability to determine whether these differences are specific to adolescents with depression or are more general adolescent risk factors.

**Table 3:** Comparison of total and factor scores of CTQ between the two groups (± s).

Item	Suicide group (N=150)	Non-suicide group (N=104)	t	P
CTQ Total Score	51.21 ± 12.71	44.53 ± 11.51	4.261	<0.01
Emotional Neglect	15.93 ± 4.69	14.06 ± 4.51	3.170	<0.01
Physical Neglect	9.79 ± 3.33	8.35 ± 3.23	3.424	<0.01
Emotional Abuse	9.04 ± 2.78	7.81 ± 2.78	3.465	<0.01
Physical Abuse	7.97 ± 3.52	6.96 ± 2.96	2.392	0.018
Sexual Abuse	8.48 ± 3.45	7.36 ± 2.46	2.827	<0.01

### 3.4 Correlation analysis between suicidal behavior in adolescents with depression and each factor of SSMSS and CTQ

Suicidal behavior in adolescents with depression was positively correlated with emotional neglect, physical neglect, emotional abuse, physical abuse, sexual abuse, teacher pressure, family environment, parental upbringing, friends and classmates, social culture, and self-physical and mental scores (r1 = 0.200, r2 = 0.229, r3 = 0.181, r4 = 0.210, r5 = 0.228, r6 = 0.153, r7 = 0.181, r8 = 0.170, r9 = 0.206, r10 = 0.184, r11 = 0.209; P<0.05).

### 3.5 Logistic regression analysis of suicidal behavior in adolescents with depression

Binary logistic regression analysis was performed with suicidal behavior in adolescents with depression as the dependent variable and age, gender, alcohol consumption, each factor score of the SSMSS scale, and each factor score of the CTQ scale as independent variables. The results showed that lower age, poor interpersonal relationships, presence of emotional abuse, and alcohol consumption are risk factors for suicidal behavior in adolescents with depression (P<0.05) as shown in **Table 4**.

**Table 4:** Multivariate linear stepwise regression analysis of suicidal risk in adolescents with depression.

Item	*B*	*SE*	*Wald*	*P*	*OR*	95%*CI*	
						*L*	*U*
Age	-0.226	0.082	7.716	0.005	0.797	0.680	0.935
Friends and Classmates	0.055	0.024	5.189	0.023	1.056	1.008	1.107
Emotional Abuse	0.109	0.052	4.491	0.034	1.115	1.008	1.234
Drinking	0.687	0.313	4.804	0.028	1.988	1.075	3.675
Constant	1.974	1.310	2.269	0.132	7.200	–	–

## 4 Discussion

In this study, upon identifying suicidal behavior in patients, we promptly implemented a range of interventions, including Cognitive Behavioral Therapy (CBT), lithium pharmacotherapy, community and family support, safety planning, and crisis intervention, to prevent further harm to the patients.

The study revealed that adolescents with depression who engaged in suicidal behaviors had a significantly higher prevalence of alcohol consumption compared to those without such behaviors. However, the binary assessment of alcohol consumption (“yes” or “no”) does not account for frequency, quantity, or type of alcohol consumed. Future studies should incorporate more detailed questions about alcohol consumption patterns to provide a more nuanced understanding of this relationship. Alcohol consumption, a common issue during adolescence, affects a considerable number of young people, with experimentation rates up to 52.8% and actual drinking rates at 24.9%, being more common among males than females. Early onset of drinking is associated with an increased risk of self-harm, particularly among those who engage in heavy drinking and are more likely to repeatedly self-harm (13–15). The risk of suicide in individuals with depression is positively correlated with the severity of depression (16); severely depressed individuals may use alcohol to numb negative emotions, and alcohol can intensify impulsive behaviors and reduce self-control, making them more susceptible to suicidal actions under its influence (17). In clinical practice, healthcare providers should be vigilant about the potential for alcohol use to exacerbate suicidal risk in adolescents with depression. Screening for alcohol use should be thorough and include questions about frequency, quantity, and type of alcohol consumed. Additionally, clinicians should consider the possibility of bipolar disorder or other psychiatric comorbidities when assessing adolescents with depression and a history of alcohol use. Early identification and appropriate management of these conditions are essential for reducing the risk of suicidal behavior and improving overall mental health outcomes.

Research indicates that early adolescence (ages 10 to 14) is a critical period for suicidal behavior (18). Our study’s univariate analysis also showed that individuals with suicidal behaviors were younger than those without, and regression analysis confirmed a significant correlation between age and suicidal behaviors. The average age of first hospitalization for adolescents with depression is 14.7 years, with those exhibiting suicidal behaviors being even younger, possibly due to suicidal behaviors more readily drawing the attention of parents and educators, prompting timely medical intervention. The younger the individual, the poorer their self-control and emotional regulation, making them more prone to impulsive suicidal acts. This finding aligns with Qiu et al. (19), who found that self-harm and suicidal behaviors were the primary reasons for adolescent depression in psychiatric emergency settings. Adolescents, being in a developmental stage, are more sensitive to stress due to hormonal changes and have relatively weaker abilities to cope with stressors, leading to greater psychological fluctuations. Negative events from school and family life can act as stressors, triggering mental health issues in adolescents. Our study found that adolescents with suicidal behaviors experienced higher stress in multiple areas, including from teachers, family environment, parenting, peers, social culture, and self-physical and mental aspects, compared to those without suicidal behaviors, with higher stress being associated with higher suicide risk. Adolescents are prone to depression and anxiety, and even suicidal behavior, when faced with teacher ridicule, criticism, disciplinary action, injustice, family tension, family conflicts, flawed parenting, bullying by peers, disputes, and discrimination. This is consistent with the research of Li (4) and Sun (10), which showed that school bullying and lack of support from teachers and peers can easily lead to a sense of inferiority in adolescents, inducing psychological crises. Several studies have demonstrated a significant positive correlation between stress and depression, with this association being particularly pronounced among individuals with low socioeconomic status and women. The findings underscore the critical need to provide targeted mental health interventions for high-risk groups, especially in low- and middle-income countries (1). Adolescents’ interpersonal relationships are in a sensitive phase; those with good peer relationships have a strong sense of pleasure and belonging and can provide psychological support, while those with poor peer relationships are prone to feelings of loneliness and loss, or lack of peer support in the face of difficulties. Childhood peer rejection and emotional neglect can also induce suicidal ideation through depression (20, 21), leading to suicidal behavior. Family is an important environment for adolescent growth, and a good family environment is crucial for the mental health development of adolescents. Unreasonable parenting and adverse family environments may increase the risk of adolescent suicide (22, 23). Poor parent-child relationships due to parental behavior, emotional issues, and inconsistent discipline can leave adolescents without effective support in difficulties, leading to negative thoughts (24). Adolescents with depression who have suicidal behaviors have experienced more traumatic events in childhood, and emotional abuse has a predictive effect on suicidal behavior in adolescents with depression, consistent with the research results of Yang et al. (25). In terms of personal and social stress, adolescents have heavy study tasks, unclear personal role positioning, and a mentality of comparison and jealousy, and their cognition is limited, making them susceptible to psychological problems. At the same time, with the popularity of the internet and mobile terminals, adolescents are exposed to more adverse information online, increasing the risks of internet gaming addiction and online relationships (26). Under the stimulation of various stressors and without effective support from family, school, and interpersonal relationships, the risk of depression and suicide in adolescents increases.

Regression analysis indicates that adolescents with depression who are younger, have poor interpersonal relationships, have experienced emotional abuse, and engage in drinking behavior are at a higher risk of suicidal behavior. In summary, the incidence of suicidal behavior in adolescents with depression is high, and the younger the adolescent with depression, the higher the risk of suicide. Adolescents under various stressors from teachers, family, parenting, interpersonal relationships, social culture, and self-physical and mental aspects are at a higher risk of suicide. Therefore, in the treatment of adolescent depression and suicide, it is necessary to pay more attention to the stress factors in the environment where adolescents are located, and to provide psychological support for adolescents at the family, school, and social levels to ensure the mental health of adolescents. The findings of this research will not only contribute to the scientific understanding of adolescent depression and suicidal behavior but also have practical implications for the development of targeted interventions and policies aimed at protecting the mental health of adolescents. By highlighting the significance of environmental factors, this study emphasizes the need for a multifaceted approach to mental health support, involving families, educational institutions, and society at large. Through this comprehensive examination, we aim to shed light on the complex dynamics of adolescent depression and the critical importance of addressing the associated stressors to prevent suicidal behaviors.

## 5 Limitations of the study

### 5.1 Lack of a healthy control group

The absence of a healthy control group limits our ability to determine whether the identified risk factors are specific to adolescents with depression or are more general adolescent risk factors. Future studies should include a control group of healthy adolescents to provide a more comprehensive understanding of the specific impact of depression on suicidal behavior.

### 5.2 Limited information on alcohol consumption

The questionnaire used in this study only captured the presence of an “alcohol consumption history” without specifying the frequency, quantity, or type of alcohol consumed. This limitation means that the data may not fully reflect the extent to which alcohol use contributes to suicidal risk. Future research should incorporate more detailed questions about alcohol consumption patterns to provide a more nuanced understanding of this relationship.

Potential comorbidity with bipolar disorder: The study did not account for the potential presence of bipolar disorder among participants. Given that alcohol use can be a symptom of bipolar disorder and that individuals with bipolar disorder are at a higher risk of developing suicidal behaviors, it is crucial to consider this potential comorbidity in future studies. This consideration is particularly relevant because the questionnaire’s binary approach to alcohol consumption (“yes” or “no”) does not allow for a detailed assessment of the nature and extent of alcohol use, which could be indicative of underlying bipolar disorder or other psychiatric conditions.

### 5.3 Cross-sectional design

The cross-sectional nature of the study limits the ability to establish causality between the identified risk factors and suicidal behavior. Longitudinal studies are needed to better understand the temporal relationships and potential causal pathways.

### 5.4 Sample size and representativeness

The sample size, although substantial, may not be representative of the broader adolescent population with depression. Future studies should aim to include larger and more diverse samples to enhance the generalizability of the findings.

### 5.5 Self-reported data

The reliance on self-reported data for assessing suicidal behavior and stress factors may introduce biases. Adolescents may underreport or overreport their experiences, which could affect the accuracy of the findings. Future studies should consider incorporating multiple data sources, such as clinical assessments and parental reports, to validate self-reported information.

### 5.6 Selection bias

The exclusive use of hospitalized patients introduces selection bias, as this sample may not be representative of the broader adolescent population with depression. Future studies should consider including outpatients or community-based adolescents to enhance generalizability.

### 5.7 Cultural contextualization

The study population is specific to Chinese adolescents, and cultural factors may influence the expression and perception of suicidal behavior. Future studies should consider cultural adaptations of assessment tools and explore the role of cultural factors in the development of suicidal behavior among adolescents.

### 5.8 Developmental differences

The wide age range (11-18 years) spans critical developmental periods with different risk profiles. Future studies should consider analyzing data separately for early (11-14 years) and late (15-18 years) adolescence to better understand age-specific risk factors.

## Data Availability

The original contributions presented in the study are included in the article/supplementary material. Further inquiries can be directed to the corresponding author.
